# Engineered macrophage membrane‐enveloped nanomedicine for ameliorating myocardial infarction in a mouse model

**DOI:** 10.1002/btm2.10197

**Published:** 2020-11-19

**Authors:** Yugang Xue, Guangwei Zeng, Jin Cheng, Jianqiang Hu, Mingming Zhang, Yan Li

**Affiliations:** ^1^ Department of Cardiology Tangdu Hospital, Air force Military Medical University Xi'an Shaanxi China; ^2^ Section 2, Department of Cardiology Xi'An International Medical Center Hospital Xi'an Shaanxi China

**Keywords:** macrophage membrane, mice, myocardial infarction, nanomedicine

## Abstract

Myocardial infarction (MI) is the serious condition causing lots of death over the world. Myocytes apoptosis, inflammation, and fibrosis are three important factors implicated in pathogenesis of MI. Targeting these three factors has been shown to ameliorate MI and rescue cardiac function. Previous studies have demonstrated that microRNA (miR) 199a‐3p protect against MI. In this study, we prepare macrophage membrane coated nanoparticles (MMNPs) containing miR199a‐3p. We evaluate the effects of these NPs on apoptosis and cell proliferation *in vitro* and the effects on inflammation cytokine production, expression of fibrosis related proteins, cardiac injuries, and functions in MI mice. We find that the MMNPs have receptors of interleukin‐1β (IL‐1β), interleukin‐6 (IL‐6), and tumor necrosis factor alpha (TNF‐α) and can bind to these cytokines. MMNPs prevent hypoxia‐induced apoptosis and promote cell proliferation, suppress the inflammation, and inhibit the cardiac fibrosis in MI mice. These results demonstrate that MMNPs ameliorate left ventricular remodeling and cardiac functions, and protect against MI, suggesting MMNPs containing miR199a‐3p is a potential therapeutic approach to treat MI.

## 1 INTRODUCTION

Myocardial infarction (MI), also known as heart attack, is caused by decreased or stopped blood flow to the heart.^1^ MI causes injuries to the heart muscle. Because of cardiomyocytes loss, MI damages myocardial functions and finally results in heart failure, causing major death and disability worldwide.

Apoptosis and fibrosis play essential roles in MI‐induced tissue injury.^2^ Following a MI, numerous cells die in response to ischemia. Apoptosis greatly contributes to myocytes death in MI and mostly occurs in the peri‐infarcted region. Apoptosis plays a critical role in determining infarct size and early symptoms of heart failure.^3^ Inhibition of apoptosis has been shown to improve cardiac functions and decrease infarct size in MI mice model.^4^ Due to the limited capacity of heart regeneration, fibrotic scars replace the lost cells.^5^ Fibrosis is an essential process for damage repair but the accumulation of fibrosis in tissues will lead to organ dysfunction and organ failure. Although inhibiting cardiac fibrosis from progressing has been recognized as important to prevent heart failure, there is still no efficient therapy available.

Inflammation is another hallmark of MI.^6^ MI triggers inflammatory responses, which result in cytokine production and inflammatory leukocytes infiltration into myocardial region. After MI, elaborated cytokines have been identified in infarcted area. Elevated tumor necrosis factor alpha (TNF‐α), interleukin (IL)‐1, and IL‐6 have been shown to contribute to myocytes death, myocardial injury, and healing process.^6^, ^7^ Blockage of TNF‐α, IL‐1, and IL‐6 has been shown to preserve the cardiac function, indicating targeting these inflammatory cytokines should be a potential therapeutic approach to treat MI.^8^, ^9^, ^10^, ^11^

MicroRNAs (miRNAs) are short non‐coding RNAs, which regulate gene expression. MiRNAs are shown to regulate various aspects of cardiomyocyte biology.^12^ MiR‐199a‐3p has been shown to reduce the infarct size and improve cardiac function in MI mice.^13^, ^14^ Nanoparticles (NPs) have been utilized as novel approach to deliver drugs for multiple diseases treatments with higher specificity and fewer side effects. Cationic lipid‐assisted PEG‐b‐PLGA nanoparticles (CLAN), which form a clinically translatable nucleic acid delivery system, has been also widely utilized to deliver miRNA, and these NPs containing miRNA have been shown to prevent cell apoptosis and improve myocardial remodeling after MI.^15^ Recently, cell‐membrane‐enveloped NPs have been recognized as an encouraging therapeutic platform.^16^ These NPs are fused with natural cell membranes and can absorb and neutralize molecules.^17^, ^18^ The neutrophil membrane‐coated NPs have been described to neutralize pro‐inflammatory cytokines, suppress inflammation, and against joint damage.^19^ Taken together, the membrane coated NPs containing miRNA could be an efficient treatment for MI.

In the present study, we prepared NPs enveloped with membranes from engineered macrophages, which overexpressed TNF‐αR, IL‐1βR, and IL‐6R. The NPs contained miR199a‐3p, which has been shown to induce cardiomyocyte proliferation.^20^ We investigated the potential effects of these membrane envelope NPs on cell proliferation, inflammatory response, and cardiac function in MI.

## 2 RESULTS

### 2.1 Preparation and characterization of macrophage membrane‐enveloped NPs encapsulating miRNA

**FIGURE 1:**
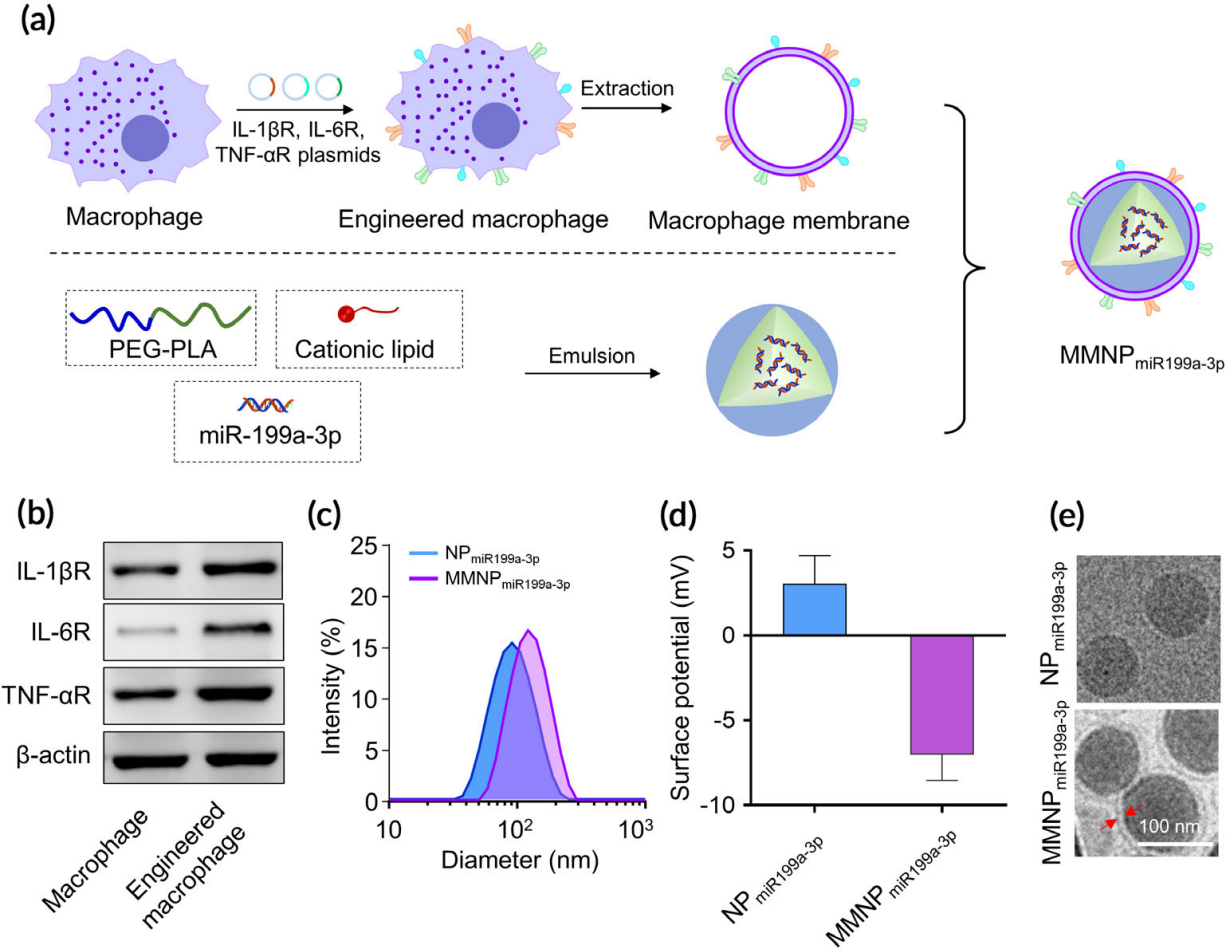
Construction of engineered macrophage membrane‐enveloped nanoparticles encapsulating miR199a‐3p for the amelioration of myocardial infarction. (a) Schematic representation of engineered macrophage membrane‐enveloped nanoparticles encapsulating miR199a‐3p. Murine macrophages were transfected with plasmids encoding IL‐1βR, IL‐6R, and TNF‐αR. MiR199a‐3p was encapsulated into polyethylene glycol–polylactic acid (PEG–PLA) via a double emulsion method, denoted as NP_miR199a‐3p_. Engineered macrophage membrane enveloped NPmiR199a‐3p was denoted as MMNP_miR199a‐3p_. The hydrodynamic size (b) and zeta potential (c) of NP_miR199a‐3p_ and MMNP_miR199a‐3p_ were examined dynamic light scattering (DLS). (d) Representative images of NP_miR199a‐3p_ and MMNP_miR199a‐3p_ examined with transmission electron microscopy (TEM). Samples were stained with uranyl acetate. Scale bar, 100 nm

First, we prepared the engineered macrophage membrane‐enveloped NPs encapsulating miR‐199a‐3p (Figure 1a). This nanoparticle was termed as MMNPmiR_199a‐3p_. The NPs encapsulating miR‐199a‐3p was denoted as NP_miR‐199a‐3p_. The engineered macrophages had enhanced surface expression of IL‐1βR, IL‐6R, and TNF‐αR (Figure 1b). We further analyzed the size and surface potential of NP_miR‐199a‐3p_ and MMNPmiR_199a‐3p_. We found membrane‐enveloped MMNPmiR_199a‐3p_ had bigger size than NP_miR‐199a‐3p_ (Figure 1c) and these two kinds of NPs had opposite surface potentials (Figure 1d). The morphology of NP_miR‐199a‐3p_ and MMNPmiR_199a‐3p_ was further analyzed by cryogenic transmission electron microscope (cryo‐TEM) (Figure 1e). The membrane envelop was observed in MMNPmiR_199a‐3p_.

#### 2.1.1 MMNP_miR199a__‐3p_ binds to inflammatory cytokines

**FIGURE 2:**
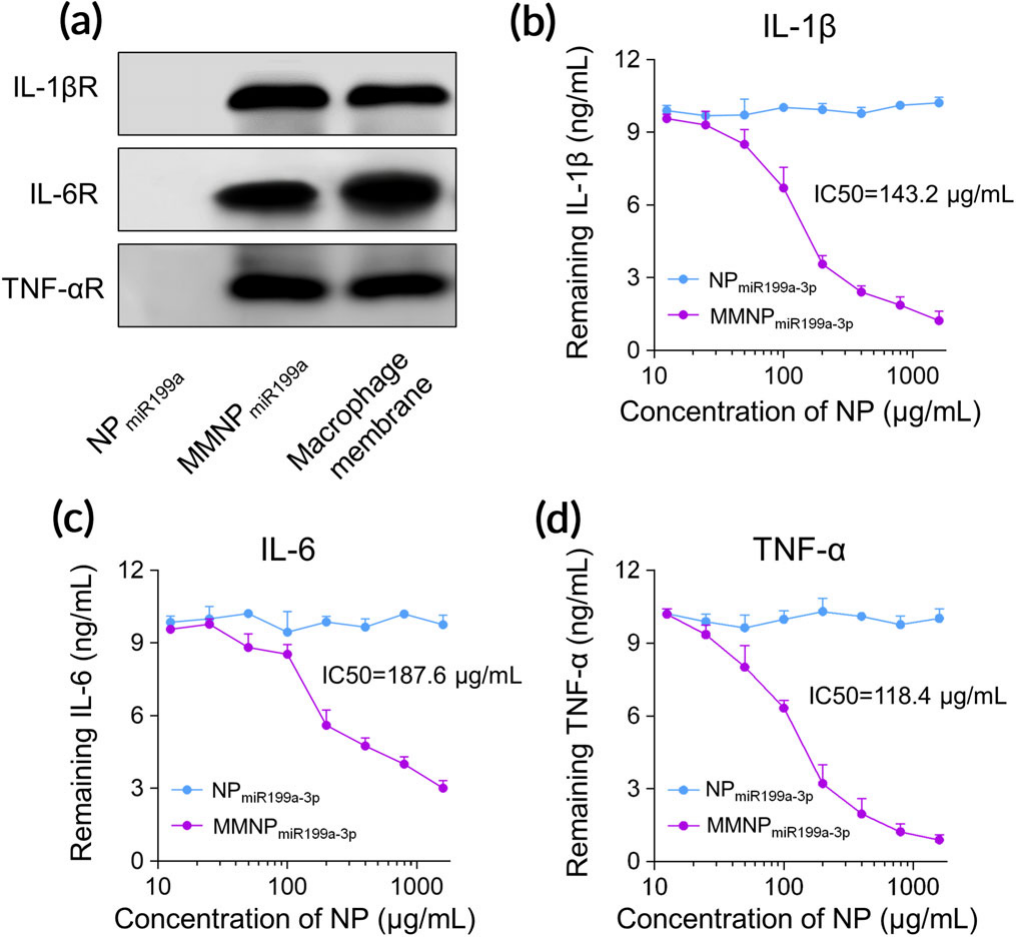
MMNP_miR199a‐3p_ can bind to inflammatory cytokines. (a) Characteristic protein bands of NP_miR199a‐3p_ and MMNP_miR199a‐3p_ examined by western blotting, macrophage membrane lysate was set as positive control. Binding capacity of MMNP_miR199a‐3p_ with IL‐1β (b), IL‐6 (c), and TNF‐α (d). Recombinant mouse IL‐1β, IL‐6, and TNF‐α (10 ng/ml) was mixed with NP_miR199a‐3p_ and MMNP_miR199a‐3p_ at final concentrations ranging from 0 to 1.6 mg/ml. The mixtures were incubated for 2 h at 37°C and nanoparticles were removed by centrifugation. The remaining proteins were examined by ELISA

Since the MMNP_miR199a‐3p_ was coated by the membrane of engineered macrophage, which had enhanced expression of IL‐1βR, IL‐6R, and TNF‐αR, we characterized the expression of IL‐1βR, IL‐6R, and TNF‐αR and potential cytokines binding ability of MMNPmiR_199a‐3p_. As shown in Figure 2a, we detected large amount of IL‐1βR, IL‐6R, and TNF‐αR in membrane fraction of macrophage as well as in MMNPmiR_199a‐3p_. In contrast, IL‐1βR, IL‐6R, and TNF‐αR were not detected in NPmiR_199a‐3p_. We continued to test the binding ability of MMNPmiR_199a‐3p_ to IL‐1β, IL‐6, and TNF‐α. We incubated IL‐1β, IL‐6, and TNF‐α with difference amounts of MMNPmiR_199a‐3p_ or NPmiR_199a‐3p_. After removal of NPs, the remaining cytokines in the supernatant were detected. As shown in Figure 2b, the level of remaining IL‐1β in the supernatant of MMNPmiR_199a‐3p_ /IL‐1β mixture decreased with increased concentration of MMNPmiR_199a‐3p,_ indicating MMNPmiR_199a‐3p_ bound to IL‐1β. In contrast, the level of remaining IL‐1β in the supernatant of NPmiR_199a‐3p_/IL‐1β mixture did not change with increased concentration of NPmiR_199a‐3p_, indicating NPmiR_199a‐3p_ did not bind to IL‐1β. Similarly, we also found only MMNPmiR_199a‐3p_ bound to IL‐6 (Figure 2c) and TNF‐α (Figure 2d).

### 2.2 NP_miR199a__‐3p_ and MMNP_miR199a__‐3p_ promoted murine cardiovascular cells proliferation under hypoxic condition

**FIGURE 3:**
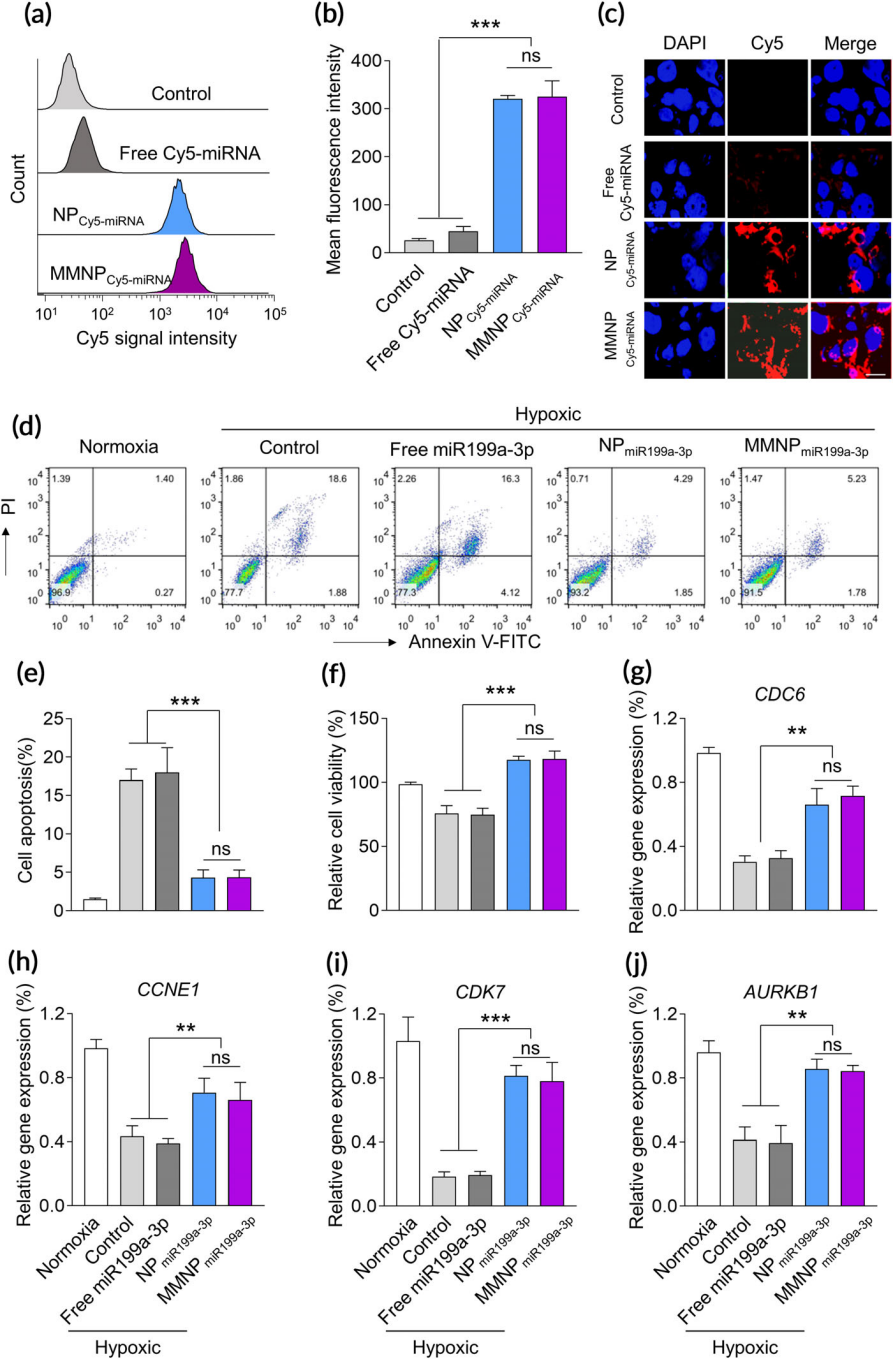
NP_miR199a‐3p_ and MMNP_miR199a‐3p_ promote the proliferation of murine cardiovascular cells under hypoxic condition. (a) Flow cytometric analysis of HL‐1 cardiac muscle cell after 4 h incubation with free or nanoparticle encapsulated Cy5‐labeled miRNA. The concentration of Cy5‐miRNA was 100 nM. (b) Mean fluorescence intensity based on the flow cytometric analysis. Data represent means ± SD. ns, no significant difference. ***p < .001. (c) Confocal microscopic images show the cellular up‐taken of NP_Cy5‐miRNA_ and MMNP_Cy5‐miRNA_. (d) Cell viability of HL‐1 cells after treated with different formulations. To generate hypoxic culture condition, HL‐1 cells culture was carried out in a hypoxic incubator containing 1% O_2_ for 6 h at 37°C. Cells were treated with free miR199a‐3p, NP_miR199a‐3p,_ and MMNP_miR199a‐3p_ (100 nM) for 24 h before hypoxic culture. Cell apoptosis and proliferation were examined after culture in hypoxic condition for 24 h. (d) and (e) Cell apoptosis were detected by flow cytometry after stained with PI and Annexin V‐FITC. (f) Cell viabilities were examined by MTT assay. The cell cycle related genes, including CDC6 (g), CCEN1 (h), CDK7 (i), and AURKB (j), were examined by real‐time PCR. Cells were treated as the MTT assay. Data represent means ± SD. ns, no significant difference. **p < .01, ***p < .001

As miR‐199a‐3p is a hypoxia‐related miRNA and has been shown to induce cardiomyocyte proliferation,^21^, ^22^, ^23^ we continued to evaluate the potential effects of NP_miR199a‐3p_ and MMNP_miR199a‐3p_ on proliferation of murine cardiovascular cells. First, we tested the uptake of Cy labeled miR‐199a‐3p by HL‐1 cells. HL‐1 cells incubated with NP_Cy5‐miR199a‐3p_ and MMNP _Cy5‐miR199a‐3p_ had significantly increased Cy5 signal (Figure 3a,b). In contrast, HL‐1 cells incubated with free Cy5 labeled miR‐199a‐3p had no Cy5 signal. These results indicated that HL‐1 cells uptook NP_Cy5‐miR199a‐3p_ and MMNP_Cy5‐miR199a‐3p_ but did not uptake free Cy5‐miR‐199a‐3p. Interestingly, we detected similar Cy5 intensity between HL‐1 cells mixed with NP_Cy5‐miR199a‐3p_ and HL‐1 cells mixed with MMNP_Cy5‐miR199a‐3p_, suggesting HL‐1 cells had similar efficacy of uptaking these two kinds of NPs. We also compared the miRNA delivery efficiency between NPs and Lipofecatime 2000 and found all delivery approach can efficiently deliver the miRNA into cells and Lipofectamine 2000 had slightly higher miRNA delivery efficacy compared with NP and MMNP. In addition, NP_Cy5‐miR199a‐3p_ and MMNP_Cy5‐miR199a‐3p_ treatment resulted in similar level of apoptosis when compared to Lipofectamine 2000, indicating NPs did not cause obvious cell toxicity (Figure S1). Correspondingly, confocal analysis confirmed the uptake of NP_Cy5‐miR199a‐3p_ and MMNP_Cy5‐miR199a‐3p_ Cy5 signal by HL‐1 cells (Figure 3c). To test the effects of NPs on apoptosis under hypoxia condition, we pretreated HL‐1 cells with NPs for 24 h and then cultured the cells under hypoxic condition. As shown in Figure 3d, we detected increased PI/Annexin V double positive cells in HL‐1 cells cultured under hypoxia condition, indicating hypoxia induced apoptosis in HL‐1 cells. Free miR199a‐3a, which was not up‐taken by HL‐1 cells, did not affect the percentage of PI/Annexin V double positive cells, indicating free miR199a‐3a did not affect hypoxia‐induced apoptosis in HL‐1 cells. In contrast, NP_miR199a‐3p_ and MMNP_miR199a‐3p_ significantly decreased the percentage of PI/Annexin V double positive cells, indicating miR199a‐3p, which was up‐taken by HL‐1 suppressed hypoxia‐induced apoptosis in HL‐1 cells. As HL‐1 cells had similar uptaking efficacy of NP_miR199a‐3p_ and MMNP_miR199a‐3p_, there was no significant difference of apoptotic cell percentage between HL‐1 cells pretreated with NP_miR199a‐3p_ and those pretreated with MMNP_miR199a‐3p_ (Figure 3e). Correspondingly, we detected significantly increased cell viability in HL‐1 pretreated with NP_miR199a‐3p_ and MMNP_miR199a‐3p_ when compared to HL‐1 cells without treatment or treated with free miR199a‐3p after hypoxia culture (Figure 3f). Hypoxia suppressed the expression of cell cycle‐related genes, including Cell Division Cycle 6 (CDC6) (Figure 3g), Cyclin E1 (CCNE1) (Figure 3h), Cyclin Dependent Kinase 7 (CDK7) (Figure 3i), and AURKB1 (Figure 3j). Free miR199a‐3p did not affect the expression of these genes. In contrast, pretreatment of NP_miR199a‐3p_ and MMNP_miR199a‐3p_ significantly increased the mRNA level of these genes, indicating miR199a‐3p ameliorated the expression of these genes in hypoxia condition.

### 2.3 MMNP_miR199a__‐3p_ suppressed inflammation in mice with acute MI

**FIGURE 4:**
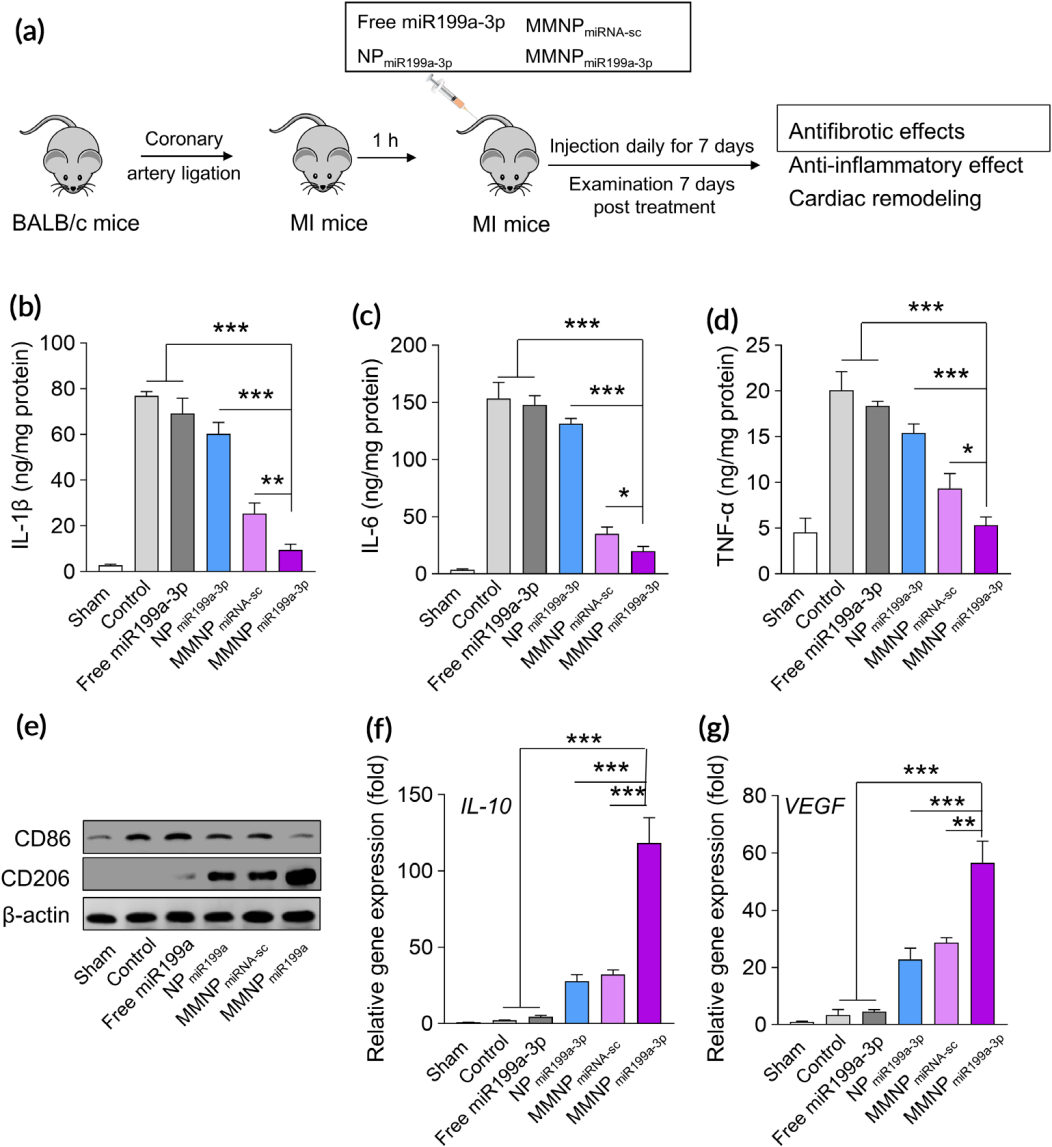
MMNP_miR199a‐3p_ treatment attenuates inflammation in vivo. (a) Schematic depicts the experiment protocol. The mice model of myocardial infarction (AMI) was established by ligating the proximal left coronary artery of male Balb/c mice for 30 minutes and then release. Mice were intravenously administrated with PBS (control group), free miR199a‐3p, NP_miR199a‐3p_, MMNP_miRNA‐sc,_ and MMNP_miR199a‐3p_, the injection dosage of miR199a‐3p and miR‐199a‐3p scramble mimic was 2.0 mg/kg, mice were treated daily for 7 days, and the antifibrotic effects, anti‐inflammatory effect and cardiac remodeling were examined two week post last injection. The concentration of IL‐1β (b), IL‐6 (c), and TNF‐α (d) in the peri‐infarct zone were detected by ELISA. (e) The expressions of CD86 and CD206 in the peri‐infarct zone two‐week post treatment were examined by western blot. The expressions of M2 macrophages related genes, including IL‐10 (f) and VEGF (g) were examined by real‐time PCR. Data represent means ± SD. *n* = 8. *p < .05, **p < .01, ***p < .001

We continued to evaluate the effects of MMNP_miR199a‐3p_ on MI *in vivo*. After intravenously administration in mice, NPs containing Cy5‐labeled miRNA can be detected in multiple organs, including the heart, suggesting NPs could display potential function in local area of heart (Figure S2). We administered free miR199a‐3p, NP_miR199a‐3p_, MMNP_miRNA‐sc_ (MMNP containing scramble miRNA), and MMNP_miR199a‐3p_ to mice with MI (Figure 4a) and evaluated the inflammatory response. MI mice had significantly increased local level of IL‐1β (Figure 4b), IL‐6 (Figure 4c), and TNF‐α (Figure 4d) in peri‐infarct tissues when compared to sham mice. Administration of free miR199a‐3p did not affect the cytokine level of IL‐1β (Figure 4b), IL‐6 (Figure 4c), and TNF‐α (Figure 4d) in peri‐infarct tissues. Administration of NP_miR199a‐3p_ slightly decreased the level of IL‐1β, IL‐6, and TNF‐α in peri‐infarct tissues, indicating the anti‐inflammatory activity of miR199a‐3p. Administration of MMNP_miRNA‐sc_ also decreased the level of IL‐1β, IL‐6, and TNF‐α, indicating the membrane associated IL‐1βR, IL‐6R, and TNF‐αR functioned to blocked the IL‐1β, IL‐6, and TNF‐α. We detected the significantly decreased level of IL‐1β, IL‐6, and TNF‐α in peri‐infarct tissues of MI mice treated with MMNP_miR199a‐3p_, indicating MMNP_miR199a‐3p_ had the best inhibitory effect on inflammation in MI mice. We further tested the expression of M1 macrophage marker CD86 and M2 marker CD206 in peri‐infarct tissues. As shown in Figure 4e, we detected increased CD86 in peri‐infarct tissues of MI mice. Administration of free miR199a‐3p did not affect CD86 expression. In contrast, administration of NP_miR199a‐3p_, MMNP_miRNA‐sc,_ and MMNP_miR199a‐3p_ decreased the CD86 expression in peri‐infarct tissues, indicating these treatments suppressed the inflammation. Correspondingly, NP_miR199a‐3p_, MMNP_miRNA‐sc,_ and MMNP_miR199a‐3p_ promoted the expression of CD206. Mice administrated with MMNP_miR199a‐3p_ had the lowest level of CD86 and the highest level of CD206 in peri‐infarct tissues, indicating MMNP_miR199a‐3p_ had the best anti‐inflammatory activities. Similarly, we detected the significantly increased mRNA level of IL‐10 (Figure 4f) and vascular endothelial growth factor (VEGF) (Figure 4g), two proteins produced by M2 macrophages, in the peri‐infarct tissues.

### 2.4 MMNP_miR199a__‐3p_ suppressed the expression of fibrosis‐related proteins

**FIGURE 5:**
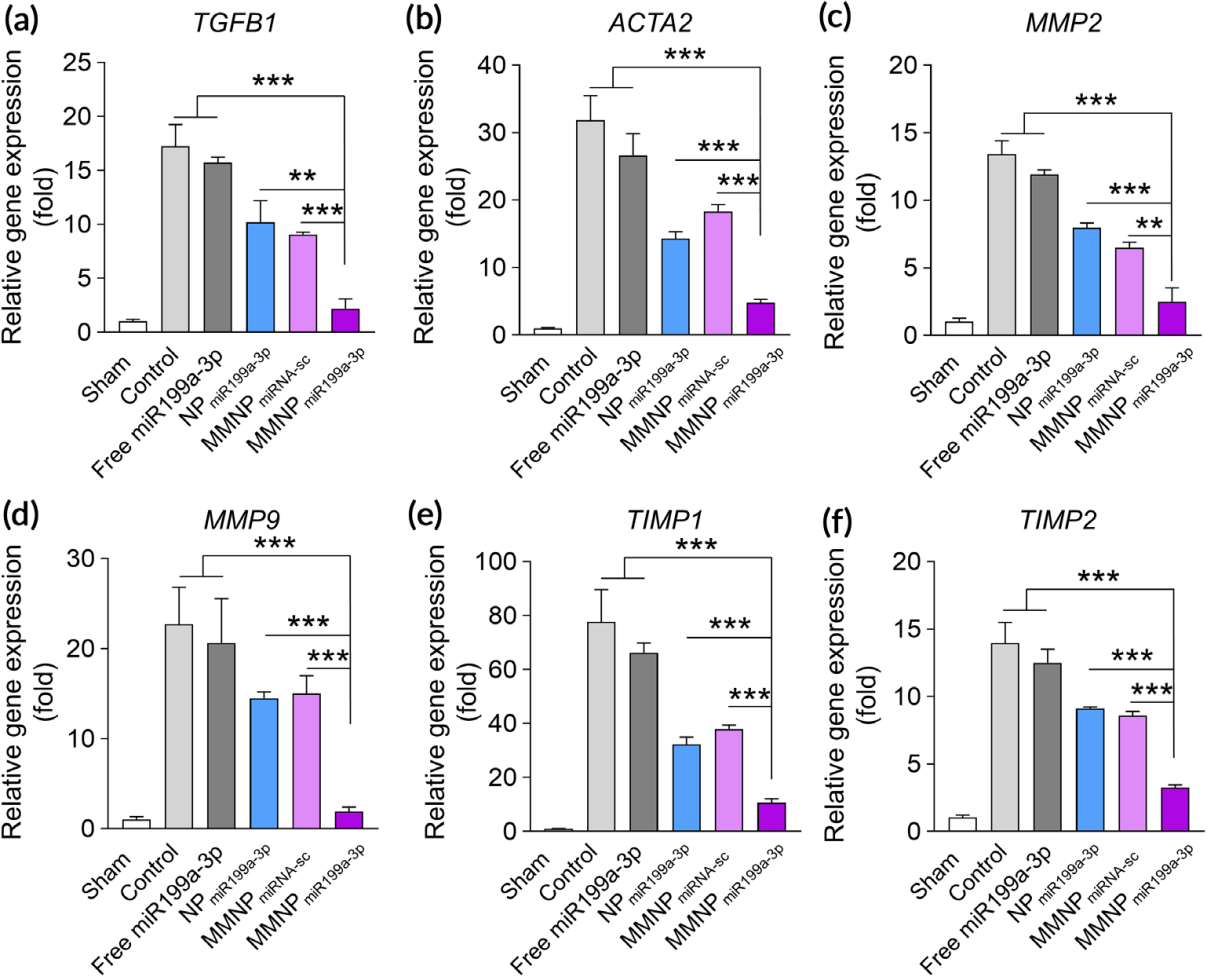
Administration of MMNP_miR199a‐3p_ inhibits expression of cardiac fibrosis markers. Relative mRNA expression levels of cardiac myofibroblast‐related genes, including TGFβ (a), ACTA2 (b), MMP2 (c), MMP9 (d), TIMP1 (e), and TIMP2 (f) in the peri‐infarct zone two week after treatment, as evaluated by real‐time PCR. Data represent means ± SD. *n* = 8. **p < .01, ***p < .001

To evaluate the effects of MMNP_miR199a‐3p_ on fibrosis in MI mice, we detected the expression level of fibrosis related genes, including TGFβ, ACTA2, MMP2, MMP9, TIMP1, and TIMP2.^24^ MI mice had elevated mRNA level of TGFβ (Figure 5a), ACTA2 (Figure 5b), MMP2 (Figure 5c), MMP9 (Figure 5d), TIMP1 (Figure 5e), and TIMP2 (Figure 5f). Administration of free miR199a‐3p slightly decreased the mRNA level of TGFβ, ACTA2, MMP2, MMP9, TIMP1, and TIMP2. NP_miR199a‐3p_ and MMNP_miRNA‐sc_ treatment also decreased the mRNA level to a lower level when compared to free miR199a‐3p. MMNP_miR199a‐3p_ treatment dramatically down‐regulated the mRNA level of TGFβ, ACTA2, MMP2, MMP9, TIMP1, and TIMP2 and the mRNA level of these genes was significantly lower than other treatment.

### 2.5 Administration of MMNP_miR199a_ protected against cardiac injury in MI mice

**FIGURE 6:**
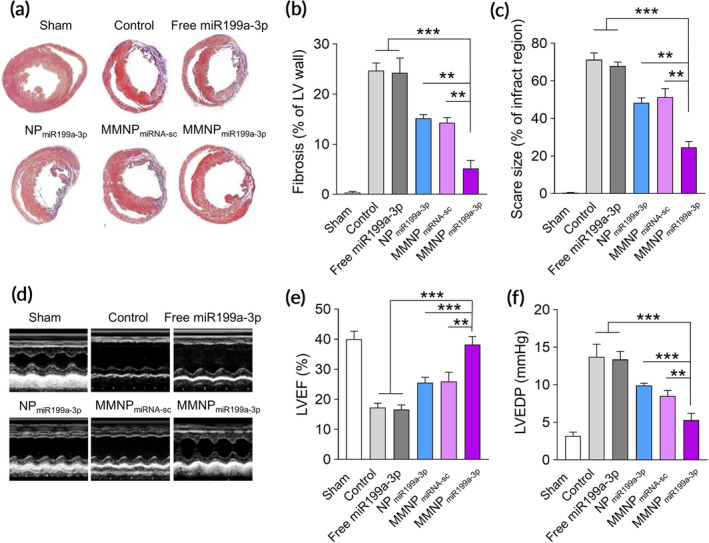
Administration of MMNP_miR199a_ improves left ventricular remodeling and promote cardiac function recovery. (a) Representative Masson's trichrome–stained infarcted hearts two weeks after treatment (blue, scar tissue; red, viable myocardium). The fibrosis area (b) and scar size (c) of the left ventricular (LV) wall one weeks after treatment. (d) Representative M‐mode images 2 weeks after treatment. (e) Left ventricular ejection fraction (LVEF). (f) Hemodynamic analyses of LV end diastolic pressure (LVEDP). Data represent means ± SD. *n* = 8. **p < .01, ***p < .001

We continued to evaluate the effects of MMNP_miR199a_ on cardiac injury and function in MI mice. We used Masson's trichrome staining to stain the transverse and coronal plane and detected obvious myocardial fibrotic area in control MI mice (Figure 6a). MI mice treated with free miR199a‐3p has similar myocardial fibrotic area to control mice. In contrast, MI mice treated with NP_miR199a‐3p_, MMNP_miRNA‐sc,_ and MMNP_miR199a‐3p_ had obvious decreased myocardial fibrotic area (Figure 6a). After quantitation, we found MMNP_miR199a‐3p_ significantly decreased both fibrosis (Figure 6b) and scare size (Figure 6c) when compared to no treatment, NP_miR199a‐3p_ or MMNP_miRNA‐sc_. In addition, MI mice had dramatically decreased LV ejection fraction while MMNP_miR199a‐3p_ treatment increased LV ejection fraction significantly more than NP_miR199a‐3p_ or MMNP_miRNA‐sc_ treatment (Figure 6d,e). Relatively, MMNP_miR199a‐3p_ treatment significantly decreased the Left ventricular end diastolic pressure (Figure 6f).

## 3 DISCUSSION

In the present study, we investigated the potential protective effects of macrophage membrane coated NPs containing miR199a‐3p (MMNP_miR199a‐3p_) on MI. The membranes were extracted from engineered macrophages overexpressing receptors of TNF‐α, IL‐1, and IL‐6. We demonstrated that the MMNP_miR199a‐3p_ absorbed inflammatory cytokines TNF‐α, IL‐1, and IL‐6, and can be efficiently up‐taken by cardiovascular cells. The up‐taken MMNP_miR199a‐3p_ prevented hypoxia‐induced cell apoptosis and promoted the proliferation of cardiovascular cells. The MMNP_miR199a‐3p_ inhibited the inflammatory response by decreasing inflammatory cytokine level in MI mice and converted a M2 phenotype environment in peri‐infarct zone. In addition, MMNP_miR199a‐3p_ prevented fibrosis, improved left ventricular remodeling, and promoted cardiac function. Therefore, these results suggested MMNP_miR199a‐3p_ could be utilized as a potential effective approach to treat MI.

Myocardial infarction is the leading cause of death. Cell apoptosis and cardiac fibrosis are two important events occur after MI. Apoptosis plays an significant role in myocardial loss after MI and is involved in the process of subsequent left ventricular remodeling and development of heart failure.^3^ In MI, dominant apoptosis is present and correlated with left ventricular remodeling.^25^ Using animal model, Bialik et al described that MI resulted in apoptosis, which only emerged in hypoxic regions during acute infarction. The hypoxia‐induced apoptosis was independent of p53.^26^ Inhibition of apoptosis has been shown to protect left ventricular function and attenuate remodeling in MI rats.^27^ Therefore, targeting apoptosis could be a useful approach to treat MI. miRNAs are small, non‐coding RNAs, which can silence gene by inhibiting mRNA translation.^28^ Increasing evidences have shown that miRNA is involved in the pathogenesis of MI.^29^ For example, he and colleagues reported that inhibition of miR‐124 inhibited cardiomyocytes apoptosis and protected against MI.^30^ Overexpressing miR‐325‐3p attenuated the cardiac tissue injury and decreased the infarct size.^31^ MiR199a‐3p has been described to regulate the cardiac cell proliferation and inducing cardiac regeneration after MI, either when expressed by adeno‐associated virus (AAV) vector^32^ or directly intra‐cardiac injection.^13^ These results suggested miR199a‐3p was a potential candidate for MI treatment. In the present study, we utilized NPs containing miR199a‐3p as the approach for treatment. We demonstrated that NPs containing miR199a‐3p (NP_miR199a‐3p_ and MM NP_miR199a‐3p_) prevented hypoxia‐induced apoptosis of cardiovascular cells, promoted the expression of cell cycle related proteins, and induced cell proliferation. Interestingly, the free miR199a‐3p, which was directly added to the cell culture had no effect on these parameters. We found that the cardiovascular cells HL‐1 cells only up‐took NPs containing Cy5‐labeled miR199a‐3p but not the free Cy5‐labled miR199a‐3p. These results strongly suggested that the correct and efficient delivery approach is one key factor in designing disease treatment. The CLAN NPs is an efficient delivery system to deliver the miRNA into target cells and enable the active function of miRNA in cells.

MI also induces inflammatory response and cytokine production. Robust production of pro‐inflammatory cytokines, including TNF‐α, IL‐1β, and IL‐6 has been detected after MI induction in rodents.^33^ The excessive inflammatory responses promote fibrosis and results in pathological remodeling and the progression of myocardial disease. Therefore, it has been suggested that inflammation could be an important target for disease preventing.^34^ Li and colleagues produced microparticles with anti‐IL‐1β antibodies and they found that these microparticles neutralized IL‐1β after MI, prevented cardiac remodeling, and induced cardiac repair.^8^ In present study, we utilized the membrane from macrophages overexpressing the receptors of TNF‐α, IL‐1β, and IL‐6 and coated to the NP_miR199a‐3p_. We found that the macrophage membrane coated NP_miR199a‐3p_ (MMNP_miR199a‐3p_) can bind to and absorb TNF‐α, IL‐1β, and IL‐6 while NP_miR199a‐3p_ cannot. In MI mice, the MMNP_miR199a‐3p_ had significantly enhanced anti‐inflammatory activities compared to NP_miR199a‐3p_, suggesting the membranes containing TNF‐αR, IL‐1βR, and IL‐6R contribute to the anti‐inflammatory activities of MMNP_miR199a‐3p_. Our findings are similar to previous study, which described that the neutrophil membrane‐coated NPs neutralized pro‐inflammatory cytokines and protected against joint damage.^19^ In the present study, delivery of NP_miR199a‐3p_ also resulted in decreased inflammation in peri‐infarct tissues, indicating miR199a‐3p itself had the anti‐inflammatory activities. Previous study by Bardin et al described that miR‐199a‐3p negatively regulated IκB Kinase β (IKKβ) expression and inhibited the nuclear factor kappa‐light‐chain‐enhancer of activated B cells (NF‐κB) signaling pathway.^35^ This result supported our finding that miR199a‐3p itself suppressed inflammation. However, the underlying mechanism of how miR199a‐3p inhibits inflammation need to be further investigated. It should be interesting to detect the NF‐κB activation in MI heart after administration of NP_miR199a‐3p_.

Several questions of current study need to be further addressed. We found the differences of anti‐inflammatory activity between NP_miR199a‐3p_ and MMNP_miR199a‐3p_. The cytokines absorption ability of MMNP_miR199a‐3p_ should contribute to the differences. It has been described that the cell membrane coated NPs is with the biological properties of the source cell from which their membrane is derived, and have prolonged circulation and disease‐relevant targeting.^36^ It is well‐known that the macrophages accumulate into heart after MI and play significantly role.^37^ It is possible that the MMNP_miR199a‐3p_ should better target to heart tissue when compared to NP_miR199a‐3p_ in MI mice while direct evidence and careful comparison are needed. In addition, to maximize the effects of NPs in MI heart, it is desired to enhance the biodistribution of NPs in MI heart. The factors involved in NPs distribution should be evaluated.

## 4 MATERIALS AND METHODS

### 4.1 Nanoparticle preparation and characterization

MiR199a‐3p, Cy5 labeled miR199a‐3p and control miRNA were obtained from RiboBio Co. (Guangzhou, China). CLAN containing miR199a‐3p was prepared using double emulsion method as described previously.^38^ Briefly, 25 mg mPEG_5K_‐b‐PLGA_11K_, 2 mg BHEM‐Chol in chloroform, and 200 mg miR199a‐3p (Ribobio, Guangzhou, China) in DNase/RNase free water were emulsified. Then another emulsion was performed. After this, the chloroform was evaporated. The NPs containing miR‐199a‐3p was termed as NP_miR199a‐3p_.

Macrophage membrane enveloped NPs were prepared as previously described.^39^ RAW 264.7 cells were transfected plasmids encoding IL‐1βR, IL‐6R, and TNF‐αR (Origene, Beijing China) using Lipofectamine LTX Reagent (Thermo Fisher, Waltham, MA) following manufacture's protocol. 48 h post transfection, cells were harvested and homogenized in hypotonic lysis buffer (20 mM Tris–HCl, 10 mM KCl, 2 mM MgCl_2_) containing protease inhibitor. After centrifuge at 3200 g for 5 min, the supernatant was saved and the pellet was re‐homogenized again. The supernatant was pooled and centrifuged at 20,000 g for 30 min at 4°C. The supernatant was ultra‐centrifuged at 80,000 g for 2 h and the pellets containing the plasma membrane were collected. Finally, the pellets were extruded through a 400‐nm polycarbonate porous membrane to get the membrane materials.

To make membrane enveloped NPs, the NPs were suspended in phosphate‐buffered saline (PBS) and mixed with prepared macrophage membranes. The mixture was then extruded. After centrifuge, the newly prepared MMNPs were suspended in PBS and stored at 4°C.

The diameters and zeta potentials of NPs were measured using Zetasizer Nano ZS90 analyzer (Malvern Panalytical Ltd, Malvern, United Kingdom). The FEI Artica Cryogenic‐Biological transmission electron microscopy (TEM) was used to detect the morphology of NPs.

### 4.2 Quantification of cytokine binding

To test the cytokine binding ability, 10 ng/ml mouse IL‐1β, IL‐6, or TNF‐α was mixed with 0 to 1.6 mg/ml NP_miR199a‐3p_ or MMNP_miR199a‐3p_. The mixtures were incubated at 37°C for 2 h. Then the NPs were removed by centrifugation at 13,000 rmp for 10 min. Cytokine concentration in the supernatant was measured using commercial mouse IL‐1β, IL‐6, or TNF‐α enzyme‐linked immunosorbent assay (ELISA) kit (Abcam, Beijing, China).

### 4.3 Cell culture

Cardiac myocyte cell line HL‐1 cells were purchased from Sigma (St. Louis, MO) and cultured in Claycomb medium supplemented with 10% heat‐inactivated fetal bovine serum (FBS), 2 mM L‐glutamine, 100 U/ml Penicillin, 100 μg/ml Streptomycin, and 100 μM norepinephrine. Cells were maintained under an atmosphere of 5% CO_2_ at 37°C. For hypoxic condition, HL‐1 cells were cultured in a hypoxic incubator containing 1% O_2_ for 6 h at 37°C.

### 4.4 MTT assay

3‐(4,5‐Dimethylthiazol‐2‐yl)‐2,5‐diphenyltetrazolium bromide (MTT, Sigma) assay was used to detect HL‐1 cell proliferation. Briefly, HL‐1 cells were seeded in 96‐well plate. After treatment, cells were treated with 100 nM free miR199a‐3p, or NP_miR199a‐3p_ or MMNP_miR199a‐3p_ containing 100 nM miR199a‐3p for 24 h and then incubated in a hypoxic incubator for 6 h. After culture for another 24 h at normal condition, 20 μl MTT reagents (5 mg/ml, Abcam, Beijing, China) was added to each well and incubated for 1 h at 37°C. The remaining crystals were dissolved in dimethyl sulfoxide. The absorbance at 492 nm was measured.

### 4.5 Apoptosis analysis

The FITC Annexin V Apoptosis Detection Kit with PI (Biolegend, San Diego, CA) was used to detect apoptosis according to manufacturer's protocols. After staining, cells were analyzed by flow cytometry using FACS Calibur (BD bioscience, San Jose, CA) and FlowJo software.

### 4.6 Reverse transcription polymerase chain reaction (RT‐PCR)

Total RNA from HL‐1 cells or tissues was extracted using RNeasy Mini Kit (Qiagen, Germantown, MD). Then the cDNA was synthesized by reverse transcription using the PrimeScript™ RT Reagent Kit (Takara, Beijing, China). Real time quantitative PCR reactions were set up with TB Green® Advantage® qPCR Premix (Takara, China) and performed using the QuantStudio 3 Real‐time PCR System (Applied biosystems). Primers used for real time PCR included: *cdc6* forward: 5′‐TCTTGACACTTCCAGTCGAAGGA‐3′, reverse: 5′‐TAGAGTCGCTG TGAGGCCACGACCACTG‐3′. *Cyclin E1* forward: 5′‐GCCAGCCTTGGGACAATAATG‐3′, reverse: 5′‐AGTTTGGGTAAACCCGGTCAT‐3′. *cdk7* forward: 5′‐AGGATGTATGGTGTAGGTGTGGA‐3′, reverse: 5′‐AAGATGTGATGCAAAGGTATTCC‐3′. *AURKB* forward: 5′‐ TCCCTGTTCGCATTCAACCT‐3′, reverse: 5′‐ GTCCCACTGCTATTCTCCATCAC‐3′. *IL‐10* forward: 5′‐AAGGCAGTGGAGCAGGTGAA‐3′, reverse: 5′‐CCAGCAGACTCAATACACAC‐3′. VEGF forward: 5′‐ GGAGATCCTTCGAG GAGCACTT‐3′, reverse: 5′‐ GGCGATTTAGCAGCAGATATAAGAA‐3′. *TGFβ* forward: 5′‐CCTGTCCAAACTAAGGC‐3′, reverse: 5′‐GGTTTTCTCATAGATGGCG‐3′. ACTA2 forward: 5′‐ACTGGGACGACATGGAAAAG‐3′, reverse: 5′‐GTTCAGTGGTGCCTCTGTCA‐3′. *MMP‐2* forward: 5′‐AAGGATGGACTCCTGGCACATGCCTTT‐3′, reverse:5′‐ ACCTGTGG GCTTGTCACGTGGTGT‐3′. *MMP‐9* forward: 5′‐AAGGACGGCCTTCTGGCACACGCCT TT‐3′, reverse: 5′‐GTGGTATAGTGGGACACATAGTGG′3′. *TIMP1* forward: 5′‐CCCAGA AATCAACGAGA‐3′, reverse: 5′‐TGGGACTTGTGGGCATA‐3′. *TIMP2* forward: 5′‐ AAGCGGTCAGTGAGAAGGAGTGG‐3′, reverse 5′‐CCTTGGAGGCTTTTTTGCAGTTG‐3′. *Glyceraldehyde‐3‐phosphate dehydrogenase (GAPDH)* forward 5′‐AGAAGGCTGGGGCTCATTTC‐3′, reverse 5′‐AGGGGCCATCC ACAGT CTTC‐3′. The relative amount of target gene mRNA was normalized to *GAPDH* mRNA. Expression levels of specific genes were normalized against the Sham group.

### 4.7 ELISA

Tissues were homogenized in tissue extraction buffer (Abcam, Beijing, China) and homogenates were centrifuged for 20 min at 13,000 rpm at 4°C. The levels of IL‐1β, IL‐6, and TNF‐α were measured using commercial ELISA kit from Abcam (Beijing, China) following manufacturer's protocols.

### 4.8 Western blot

The western blot was performed as described previously.^40^ Briefly, NPs, MMNPs, macrophage membrane extraction, or total protein extracted from heart tissues were subjected to sodium dodecyl sulfate–polyacrylamide gel electrophoresis and then were transferred to polyvinylidene fluoride membranes. The membranes were blocked with 5% non‐fat milk for overnight at 4°C. Next day, primary antibodies were added to the membrane and incubated at room temperature for 2 h. After wash, corresponding horse radish peroxidase‐conjugated secondary antibodies were incubated for 1 h at room temperature. Immuno‐reactive bands were visualized using ECL substrate kit (Abcam, China). Antibodies used in present study include: anti‐IL‐1R (Abcam, Beijing, China), anti‐IL‐6R (Abcam, China), anti‐tumor necrosis factor receptor 1 (TNFRI) (Abcam, China), anti‐CD86 (Abcam, China), and anti‐CD206 (Abcam, China).

### 4.9 Immunofluorescence assay

HL‐1 cardiac muscle cells were incubated with free or nanoparticle encapsulated Cy5‐labeled miRNA for 4 h. Then the cells were washed with PBS and fixed with 4% paraformaldehyde. The DNA was stained with 4′,6‐diamidino‐2‐phenylindole (DAPI) (Thermo Fisher) at room temperature for 5 min. After wash, cells were analyzed by confocal microscopy.

### 4.10 Mice treatment

MI was induced in Balb/c mice by coronary artery ligation as described previously.^41^ 1 h post coronary artery ligation, 2.0 mg/kg free miRNAs or NPs were injected by tail vein every day for consecutive 7 days. After treatment, tissues were harvested for analysis. The study was approved by the ethics commitment of Tangdu Hospital, Air force Military Medical University.

### 4.11 Masson staining

The fibrosis of heart was determined using Masson Trichrome Stain kit (Sigma, St. Louis, MO) following manufacturer's protocols. The collagen fibers were stained as blue and the viable myocardium was stained as red. The area of fibrosis and the scare size was quantitated using ImageJ software.

### 4.12 Echocardiography and hemodynamic study

Mice were anesthetized by isoflurane and then M‐mode images were obtained by using Visualsonics Vevo 770 echocardiography machine (Visualsonics Inc., Toronto, Canada) as described previously.^42^ Hearts areas between two papillary muscles were viewed. LV internal dimensions (LVID) at diastole (LVIDd) and systole (LVIDs) were measured. The LV ejection fraction (EF) was calculated by the software with the machine. The cardiac hemodynamic function was evaluated using a Millar tip‐pressure catheter as described previously.^42^ LV end‐diastolic pressure (LVEDP) was measured by catheter advancement into the LV cavity. Data were analyzed using the PowerLab System.

### 4.13 Statistical analysis

All data are shown as means ± standard deviations (SD). Statistical analysis was performed by one‐way ANOVA followed with a Tukey's post hoc test. *p* < .05 was considered statistically significant.

## CONFLICT OF INTEREST

No conflicts of interest, financial, or otherwise, are declared by the authors.

## AUTHOR CONTRIBUTIONS

**Yugang Xue:** Data curation; investigation; software; supervision; validation. **Guangwei Zeng:** Data curation; formal analysis; resources. **Jin Cheng:** Data curation; formal analysis; funding acquisition. **Mingming Zhang:** Supervision; validation; writing‐original draft; writing‐review and editing. **Jianqiang Hu:** Methodology; validation; visualization. **Yan Li:** Funding acquisition; resources; supervision; writing‐original draft; writing‐review and editing.

### PEER REVIEW

The peer review history for this article is available at https://publons.com/publon/10.1002/btm2.10197.

## Supporting information


**Appendix S1**: Supporting Information.

## Data Availability

Data could be obtained upon reasonable request to the corresponding author.
