# Supplementary material for: Engineered macrophage membrane‐enveloped nanomedicine for ameliorating myocardial infarction in a mouse model
Source: Bioeng Transl Med. 2020 Nov 19;6(2):e10197. doi: 10.1002/btm2.10197 (PMC8126824; doi:10.1002/btm2.10197)
Supplement: Supplementary file 1 — Appendix S1: Supporting Information. [file BTM2-6-e10197-s001.docx]

**Supplementary materials**

**Engineered macrophage membrane-enveloped nanomedicine for ameliorating myocardial infarction in a mouse model**

Yugang Xue^1^, Guangwei Zeng^2^, Jin Cheng^1^, Jianqiang Hu^1^, Mingming Zhang^1^, Yan Li^1, *^

^1^Department of Cardiology, Tangdu Hospital, Air force Military Medical University, Xinsi Road, Baqiao District, Xi’an 710000, Shaanxi, China

^2^Section 2, Department of Cardiology, Xi’An International Medical Center Hospital, No. 777 Xitai Road, Chang’an District, Xi’an 710100, Shaanxi, China

^*^Corresponding author

Yan Li

Department of Cardiology, Tangdu Hospital, Air force Military Medical University, Xinsi Road, Baqiao District, Xi’an 710000, Shaanxi, China

Email: profleeyan@163.com

Tel: 86-13892890227


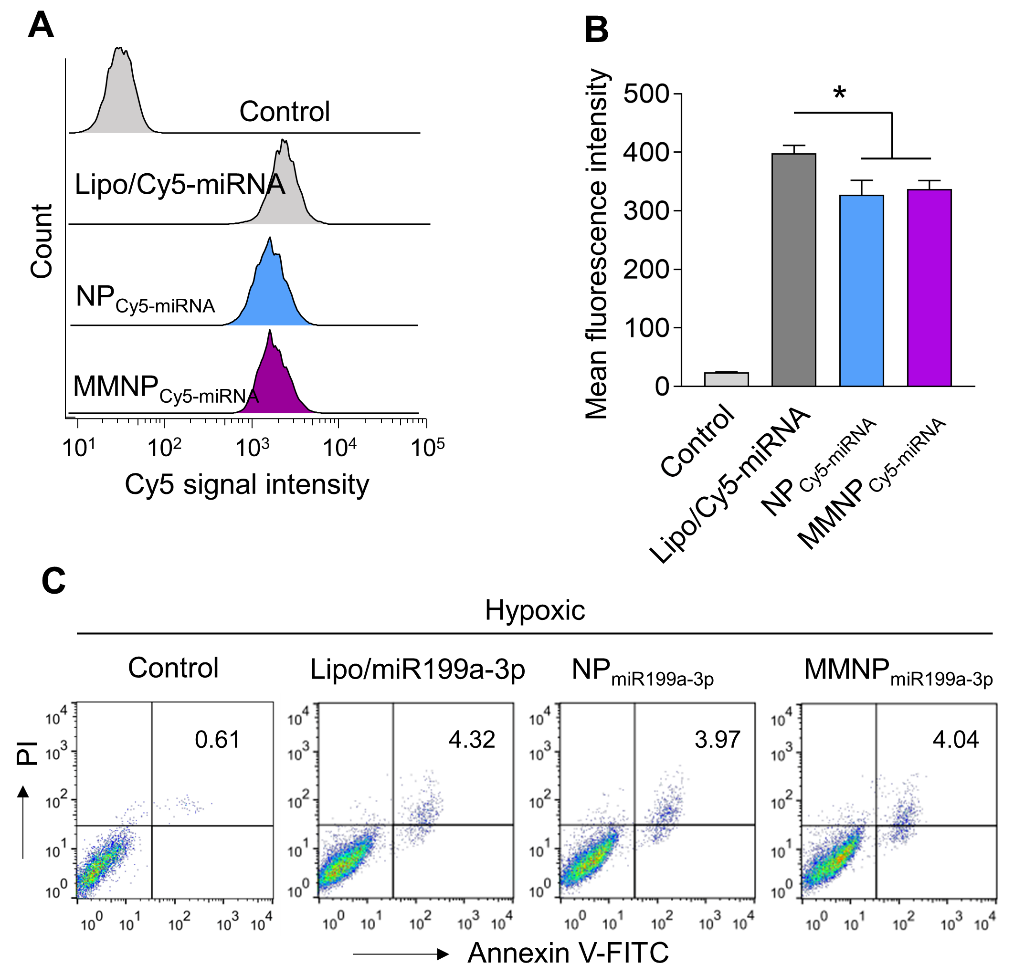


**Figure S1.** (**A**) Flow cytometric analyses of HL-1 cardiac muscle cell after 4 h incubation with Lipofectamine 2000 or nanoparticle carried Cy5-labeled miRNA. The concentration of Cy5-miRNA was 100 nM. (**B**) Mean fluorescence intensity based on the flow cytometric analysis. Data represent means ± SD. **p* < 0.05. (**C**) Cell apoptosis of HL-1 cells after treated with different formulations. To generate hypoxic culture condition, HL-1 cells culture was carried out in a hypoxic incubator containing 1% O_2_ for 6 hours at 37°C. Cells were treated with Lipo/miR199a-3p, NP_miR199a-3p_ and MMNP_miR199a-3p_ (100 nM) for 24 h before hypoxic culture. Cell apoptosis was examined after culture in hypoxic condition for 24 h.

**Figure S2.** MI mice were intravenously administrated with MNP_miR199a-3p_ or MMNP_miR199a-3_p, miR199a-3p was labeled with Cy5 and the injection dose was 2.0 mg/kg. 24 h later, mice were sacrificed and main organs (including heart, lung, liver, spleen, and kidney) were collected for examination using *in vivo* imaging system (IVIS).
